# The prevalence of low-level viraemia and its association with virological failure in people living with HIV: a systematic review and meta-analysis

**DOI:** 10.1080/22221751.2024.2447613

**Published:** 2024-12-27

**Authors:** Shengnan Zhao, Wenjing Wang, Sibo Li, Jiaze He, Wenshan Duan, Zhen Fang, Xiaoran Ma, Zhen Li, Caiping Guo, Wen Wang, Hao Wu, Tong Zhang, Xiaojie Huang

**Affiliations:** a Clinical and Research Center for Infectious Diseases, Beijing Youan Hospital, Capital Medical University, Beijing, People’s Republic of China; b School of Life Sciences, Tianjin University, Tianjin, People’s Republic of China; c Beijing Key Laboratory for HIV/AIDS Research, Clinical and Research Center for Infectious Diseases, Beijing Youan Hospital, Capital Medical University, Beijing, People’s Republic of China

**Keywords:** HIV, low-level viraemia, prevalence, risk factors, virological failure

## Abstract

Low-level viraemia (LLV) following antiretroviral therapy (ART) in people living with HIV (PLWH) has not received sufficient attention. To the determine the prevalence of LLV and its association with virological failure (VF), we systematically reviewed evidence-based interventions for PLWH. We searched PubMed, the Cochrane Library, Embase, and Web of Science from inception to 22 May 2024. Cohorts with samples sizes smaller than 1000 in size were excluded. Data from 16 cohort studies, encompassing 13,49,306 PLWH, revealed a pooled prevalence of LLV of 13.81%. Relative risk (RR) and 95% confidence intervals (CI) identified the following risk factors for LLV: viral load (VL) ≥ 10^5^ copies/mL at baseline (1.79, 1.11–2.88), AIDS-defined illness at baseline (1.24, 1.10–1.40), and protease inhibitor-based regimen at ART initiation (1.53, 1.45–1.62) are the risk factors for LLV. Conversely, CD4 count ≥200 cells/μL at baseline (0.90, 0.82–0.98), non-nucleoside reverse transcriptase inhibitor-based regimen (0.81, 0.68–0.96) and the integrase strand transfer inhibitor (INSTI)-based regimen (0.60, 0.42–0.85) were associated with a reduced risk of LLV. Pooling the adjusted hazard ratio (aHR) and the 95% CI, we found that LLV increased the risk of VF with rising VL among 96,711 PLWH (aHR 2.77, 95% CI 2.03–3.76) and increased the risk of all-cause mortality at high VL levels among 14,229 PLWH (aHR 1.66, 95% CI 1.16–2.37). Therefore, the prevalence of LLV in PLWH should not be overlooked. This study aims to guide better management strategies to improve clinical outcomes in patients with LLV.

## Introduction

Eradicating acquired immunodeficiency syndrome (AIDS) is one of the United Nations’ Sustainable Development Goals for 2030 [1]. Significant progress has been made in the fight against HIV and AIDS in recent years. Then “Undetectable = Untransmittable,” (U = U), has been increasing recognized by international organizations and countries. An HIV viral load (VL) below 200 copies/mL is associated with zero risk of sexual transmission, a threshold widely used for “U = U” messaging in many high-income settings [2]. However, in resource-limited settings, the limitations of VL testing methods do not allow for this level of surveillance [3,4]. Previously, the risk associated with VL >200 copies/mL has been debated. A systematic assessment presented at the 2023 International Antiviral Society Conference revealed minimal risk of sexual transmission among people living with HIV (PLWH) who have low-level viraemia (LLV) (<1000 copies/mL) [5]. This finding further underscores the importance of “U = U” in public health policy and practice. This provides a strong scientific basis for global HIV prevention and control. However, this encouraging conclusion does not extend to other transmission routes, such as mother-to-child transmission and injection drug use. If VL in the blood exceeds 12.7 copies/mL, there is a risk of HIV transmission through only 22.9 mL of blood transfusion [6]. The World Health Organization (WHO) classifies HIV transmission risk into three levels, with VL <1000 copies/mL categorized by “yellow light” risk [7]. The presence of LLV impedes HIV elimination.

Previous research has indicated that LLV, particularly persistent LLV (pLLV), may lead to several adverse outcomes, including the development of drug resistance mutations, increased inflammatory factors release, elevated immunological activation, and accelerated disease progression [8–12]. The definitions of LLV and virological failure (VF) and are inconsistent across current research. Therefore, the existing evidence remains controversial and cannot be applied universally.

Typically, LLV is characterized by detectable viraemia below the threshold of VF. The WHO defines VF as a first VL >1000 copies/mL 6 months after initiating antiretroviral therapy (ART), followed by a repeat VL result >1000 copies/mL 3 months after the first VL result on a global scale [13]. In contrast, high-income settings often use a threshold of 200 copies/mL [14,15], while the European AIDS Clinical Society (EACS) strictly defines VF as VL ≥50 copies/mL [16]. Due to these differing definitions, existing research evidence cannot be applied to all PLWH with LLV, and no international guidelines have been established.

Considering these discrepancies and the lack of robust evidence for lower threshold, we summarized the prevalence and risk factors for LLV within the range of 50–999 copies/mL. Additionally, we reviewed the available evidence to assess the effect of LLV on the risk of VF and mortality.

## Methods

### Search strategy and selection criteria

This systematic review and meta-analysis followed the Preferred Reporting Items for Systematic Reviews and Meta-Analysis (PRISMA) statement [17]. We searched PubMed, the Cochrane Library, Embase, and Web of Science from inception to 22 May 2024, without language restrictions to identify relevant full-text studies. Details of the search strategies are provided in Table S1. In Brief, combinations of search terms related to HIV or AIDS, LLV, pLLV, blip, and residual viraemia were used. The study protocol is registered in PROSPERO (CRD42023410779).

Clinical trials and prospective or retrospective cohort studies had to meet all of the following criteria to be included in this review: the study population must be at least 15 years of age, including PLWH with ART-naïve or ART-experienced and at risk of LLV after at least 24 weeks of ART; the study population was followed up for LLV following ART (based on first-and second-line ART); and the prevalence rate was either reported or calculated. Samples with a size of less than 1000 were excluded.

The primary outcome was LLV prevalence. The risk factors for LLV and the correlation between LLV and VF were the secondary outcomes. All participants were categorized by their longitudinal viraemia profiles six months or more after ART initiation the following definitions: (1) LLV, defined as the occurrence of at least one VL measurement of 50–999 copies/mL after virologic suppression is achieved, including pLLV, defined as two or more consecutive VLs of 50–999 copies/mL, at least one month apart, and otherwise blip; (2) VF, defined as one or more HIV VLs of ≥1000 copies/mL; and (3) virological suppression, defined as VL <50 copies/mL.

### Data analysis

The prevalence of LLV was calculated for each included study using the total number of PLWH in the cohort and the number of PLWH with LLV. The computed and pooled relative risk (RR) and 95% confidence intervals (CI) were used to evaluate LLV risk variables. If available, we recorded the adjusted hazard ratio (aHR), 95% CI, and *p*-values for the association between LLV and VF according to the most adjusted model results. To compare the syntheses, we also complied frequently published unadjusted model results. We noted the factors for which adjustments were performed in each model to provide information for evaluating residual confounding factors. To reduce meta-analysis heterogeneity, we chose the follow-up time points that were most frequently reported across trials where an outcome was reported throughout a range of time points. We performed a single-proportion meta-analysis on the prevalence of LLV. Correlation analyses employed log-transformed rates that were subsequently back-transformed for reporting purposes.

Data extracted included the first author's name, publication year, study design, study location, inclusion and exclusion criteria, definition of LLV, source of the LLV cohort, total number of participants, and PLWH demographics and clinical characteristics, such as age, sex, baseline CD4 count, baseline HIV VL, initial ART regimen, duration of follow-up, adjusted variables, and pertinent outcomes. The risk of bias in the included studies was assessed using a modified Newcastle-Ottawa quality assessment scale for cohort studies [18]. It consists of eight questions divided into three domains: Outcome (three questions), Exposure (two questions), and Selection (three questions). The total score was nine. One point was considered a moderate risk of bias for each domain, whereas more than one point was regarded as a high risk of bias. Three reviewers (SNZ, WJW, and SBL) performed critical evaluations, and discrepancies were discussed with the group to obtain agreement. Each outcome was reported only once if it appeared in multiple publications that reported outcomes in the same cohort.

We examined the subgroup proportions of PLWH with LLV according to study type, study site, and national economic status. Sensitivity analyses were conducted to evaluate differences in the proportion of PLWH with LLV. Considered significant heterogeneity was determined as *p*-value of Q statistic ≤0.1 or *I^2^* ≥ 50%. Rate estimates were compiled using Stata's metan command and either random- or fixed-effect meta-analysis models by constructing funnel plots and calculating Egger’s test with Stata’s metafunnel and metabias commands [19,20]. All data were examined using Stata SE (version 15.0).

## Results

**Figure 1.**
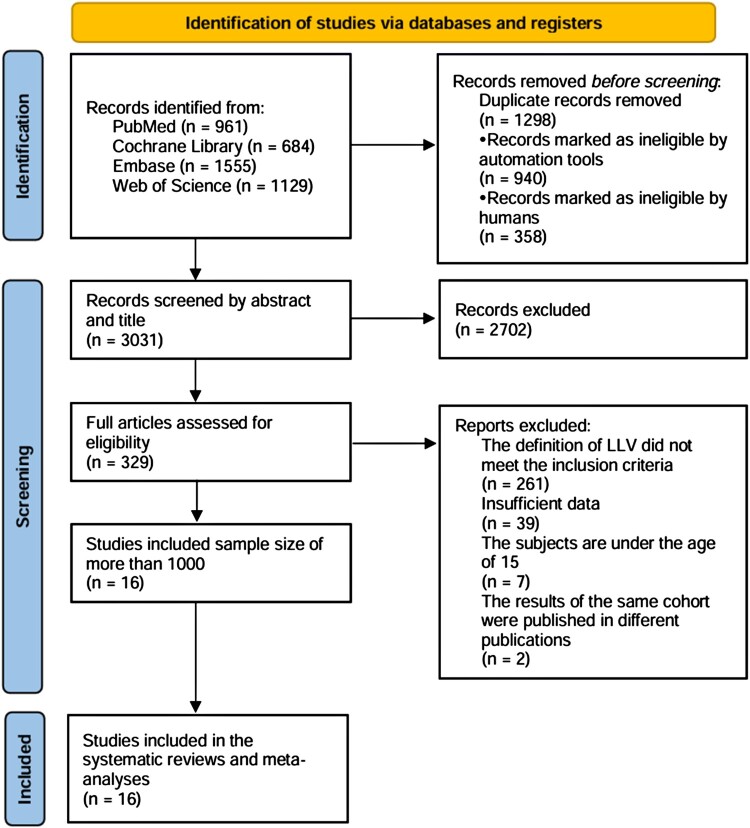
PRISMA flowchart of study inclusion. Abbreviations: PRISMA, Preferred Reporting Items for Systematic Reviews and Meta-Analyses; LLV, low-level viraemia.

The first search yielded 4,329 results; 1,298 duplicates and 2,702 ineligible titles and abstracts were excluded. From the remaining 329 articles that underwent a full-text review, we excluded three articles from the same cohort [21–23]. Eventually included 16 cohort studies with data on 13,49,306 PLWH on the prevalence of LLV (Figure 1) [24–39]. Six of these studies provided aHR and 95% CI in LLV and VF, including 1,10,219 PLWH [24,25,29,31,34,37].

The earliest study on this topic was conducted in 2012 [24]. The study designs included three prospective observational studies and thirteen retrospective observational studies. Fourteen studies reported the sex ratio of PLWH [24–27,29–38], revealing that the majority of PLWH were male, with only two studies reporting a majority of female PLWH [29,38]. The proportion of PLWH with a CD4 count <200 cells/µL varied from 12.8 to 51% across eight studies [24,26,29,31,32,34–36]. The longest follow-up period was 81,837 person-years (PYS) [37]. Hermans et al. reported the highest incidence rate [29]. The incidence rate of LLV in the first-line ART regimen was 11.5/100 PYS (95% CI 11.4–11.7), and that of the second-line ART regimen was 15.1/100 PYS (14.2–16.1) (Table S2).

The estimated risks of bias are presented in Table S3. There was a low risk of bias in five studies, moderate risk in eight studies, and high risk in three studies. The symmetry could not be assessed using funnel plots. Egger's (*p* = 0.097) and Begg's (*p* = 0.620) tests indicated no potential publication bias (Figure S1).

**Figure 2.**
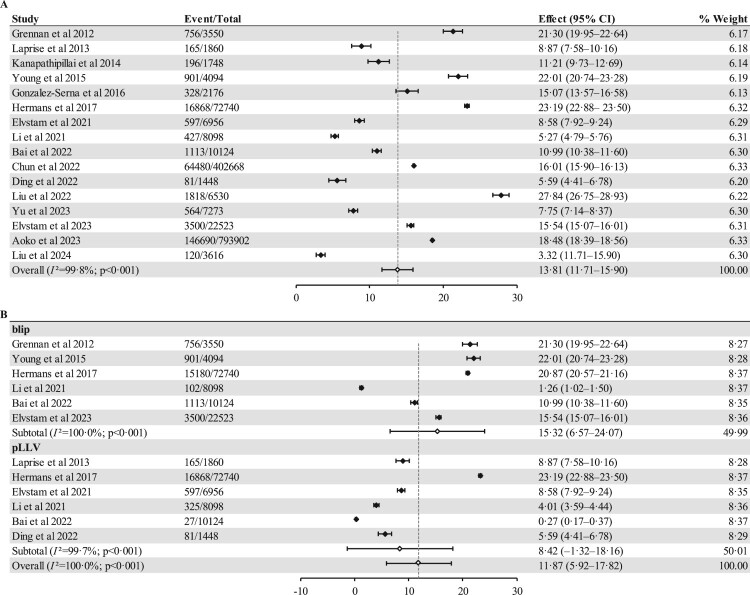
Forest plot of prevalence among PLWH with LLV (A) and among PLWH with blip or pLLV (B). Abbreviations: CI, confidence interval; LLV, low-level viraemia; pLLV, persistent low-level viraemia; PLWH, people living with HIV.

**Figure 3.**
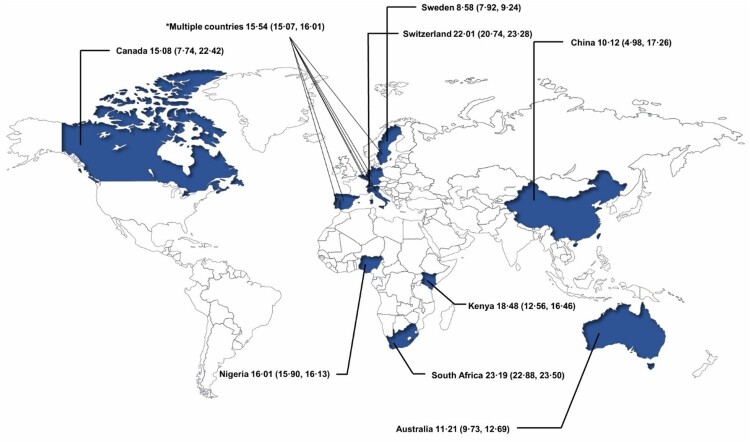
Summary of LLV prevalence in included studies. *Multiple countries: Spain, Italy, Sweden, Germany, Luxembourg, Portugal, and Belgium.

The 16 included studies provided prevalence data for LLV, resulting in a pooled prevalence of 13.81% (95% CI 11.71%–15.90%, 238,604/13,49,306). Six studies reported the prevalence of blip [24,27,29,31,32,37] while another six reported the prevalence of pLLV [25,29–32,34]. Additionally, the prevalence of blip in PLWH was substantially higher than that of pLLV (15.32%, 6.57%–24.07%, 21,552/1,21,129 vs. 4.85%, 3.25%–6.45%, 2,883/1,01,226; *p* = 0.000) (Figure 2). We summarized the LLV prevalence reported in the included studies, with LLV prevalence rates in South Africa and Switzerland at 23.19% (22.88%–23.50%) and 22.01% (20.74%–23.28%), respectively (Figure 3). Among the continents, Africa had the highest prevalence rate of 19.22% (16.59%–21.85%, 2,28,038/12,69,310) (Figure S2). In the subgroup analysis, the prevalence differed according to study site. The pooled prevalence of LLV in single-centre studies (8.15%, 5.07%–11.23%, 2,234/27,322) was significantly lower than that in the multicentre studies (17.17%, 15.27%–19.07%, 2,36,370/13,21,984). Data from studies of different types and income countries showed that the pooled prevalence of LLV was similar (Figure S3).

**Figure 4.**
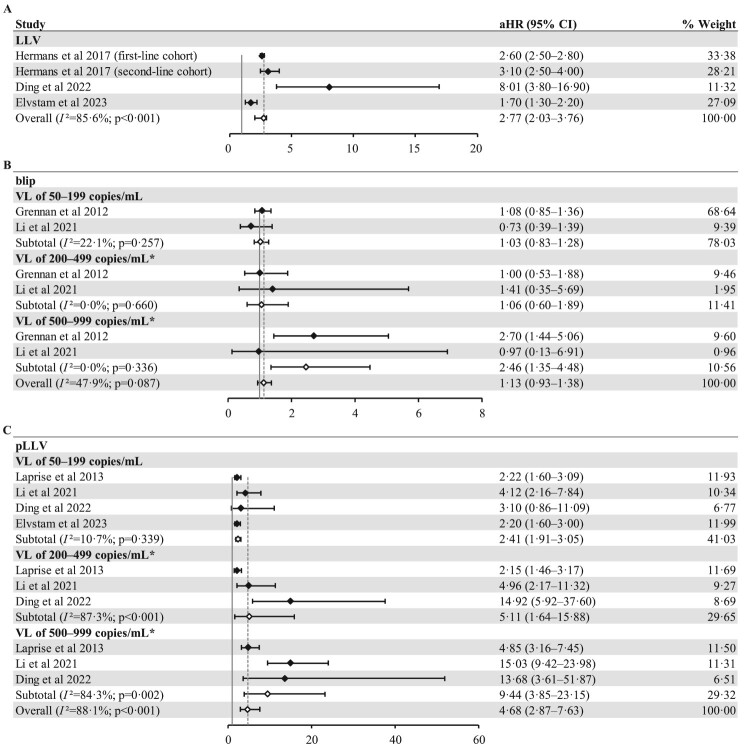
Pooled aHR for assessing the association between the risk of VF and LLV (A), blip (B), and pLLV (C). Abbreviations: aHR, adjusted hazard ratio; CI, confidence interval; LLV, low-level viraemia; pLLV, persistent low-level viraemia; VL, viral load. *Li et al. `s standard for stratified LLV is different from other articles.

Six articles, including 1,10,219 PLWH from seven cohorts, provided aHR and 95% CIs for LLV and VF [24,25,29,31,34,37]. Although the standard for stratifying LLV by Li et al. was slightly different from that used in other studies, we believe that this difference was negligible [31]. Information on each model adjustment variable is presented in Table S4. Three articles, including four cohorts with a total of 96,711 individuals, studied the association of LLV with VF, and we pooled their results ultimately finding that LLV was associated with a significantly increased risk of subsequent VF with an aHR of 2.77 (2.03–3.76) (Figure 4(A)) [29,34,37]. PLWH with a VL of 500–999 copies/mL in the blip subgroup were considerably more likely to experience VF (2.46, 1.35–4.48). The prevalence of VF was not significantly higher in PLWH in the 50–199 copies/mL and 200–499 copies/mL VL groups (1.03, 0.83–1.28; 1.06,0.60–1.89) (Figure 4(B)). The aHRs in the pLLV subgroup increased as the LLV range increased from 51 to 199 copies/mL, 200–499 copies/mL, and 500–999 copies/mL. The risk of VF was significantly greater in PLWH with VL of 50–199 copies/mL (2.41, 1.91–3.05), 200–499 copies/mL (5.11, 1.64–15.88), and 500–999 copies/mL (9.44, 3.85–23.15) than in those with virological suppression (Figure 4(C)). Similarly, the results of pooling aHR and 95% CI of six articles were 2.73 (2.02–3.68), and regardless of the level of VL, LLV was significantly correlated with VF (Figure S4). Similar results were obtained using the unadjusted model (Figure S5).

Of the 16 studies included, only one reported an aHR of 2.2 (1.3–3.6) for all-cause mortality in participants with LLV of 50–999 copies/mL compared to those with virological suppression [30]. When analysing the LLV groups separately, two studies included data from 1,429 PLWH [30,36]. Among those with 50–199 copies/mL, no significant increase in the risk of all-cause mortality (1.45, 0.67–3.13) was observed, while the aHR of 200–999 copies/mL of PLWH was 1.66 (1.16–2.37) (Figure S6).

Two studies have reported a correlation between LLV and AIDS-related death [30,36]. Yu et al. found that LLV could increase the risk of AIDS-related death at 200–999 copies/mL (2.37, 1.36–4.14), but there was no significant change in AIDS-related death risk at 50–199 copies/mL (1.12, 0.62–2.04) [36]. Two studies have reported a correlation between LLV and non-AIDS events (NAEs) [30,34]. Elvstam et al. found a significant correlation between pLLV and NAEs in pLLV individuals with VL between 200 and 999 copies/mL in the adjusted model (2.0, 1.2–3.6) [30]. Ding et al. reported that pLLV can lead to NAEs regardless of the VL level (8.39, 4.07–17.30) [34]. One study reported the drug resistance (DR) of LLV [35]. Among 1,818 PLWH with VLs of 50–999 copies/mL, 182 (10.0%) developed HIV DR. The most frequently occurring resistance-associated mutations were M184I/V (28.6%), K103N (19.2%), and V181C/I/V (10.4%), with multidrug resistance observed in 27.5% of cases.

**Figure 5.**
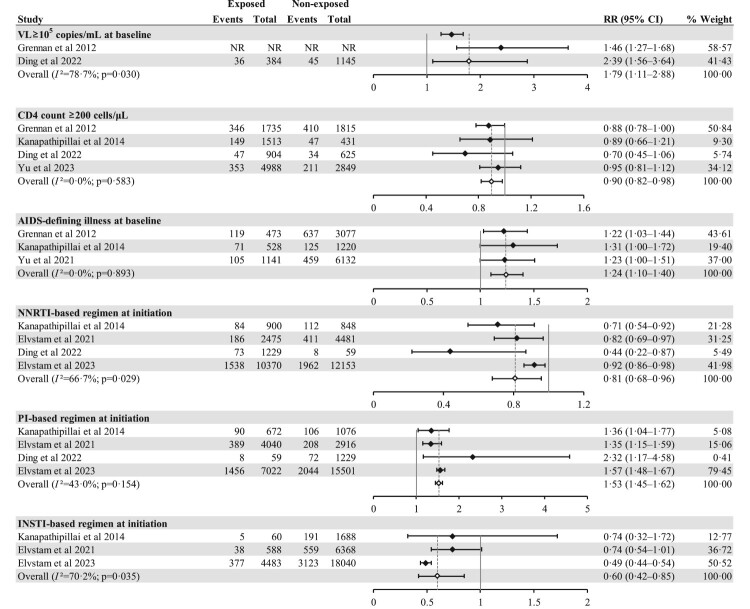
Forest plot displaying risk factors of LLV. Abbreviations: RR, risk ratio; CI, confidence interval; VL, viral load; NR, not reported; NNRTI, nonnucleoside reverse transcriptase inhibitor; PI, protease inhibitor; INSTI, integrase strand transfer inhibitors; LLV, low-level viraemia.

In the risk factor analysis of LLV, seven studies provided univariate analysis data [24,26,30,34–37]. We identified that VL ≥10^5^ copies/mL at baseline (RR 1.79, 1.11–2.88), AIDS-defined illness at baseline (1.24, 1.10–1.40), and protease inhibitor (PI)-based regimen at ART initiation (1.53, 1.45–1.62) can increase the risk of LLV. Conversely, CD4 count ≥200 cells/μL at baseline (0.90, 0.82–0.98), non-nucleoside reverse transcriptase inhibitor (NNRTI)-based regimen (0.81, 0.68–0.96) and integrase strand transfer inhibitor (INSTI)-based regimen (0.60, 0.42–0.85) appeared to reduce the risk of LLV (Figure 5). One of the studies reported that any NRTI resistance mutation detected pre-ART serves as a protective factor (0.65, 0.52–0.81) [37], while another study reported that missed doses in the past month could increase the risk of LLV (1.62, 1.49–1.75) [35]. We found no significant influence of sex, route of HIV acquisition, coinfection of hepatitis B or hepatitis C at baseline or haemoglobin levels ≥110 g/L on the outcomes (Figure S7).

## Discussion

There persistence of LLV poses a significant challenge to achieving full viral suppression in PLWH complicating efforts to control HIV epidemic. The lack of standardized definition of LLV across different guidelines, along with variations in study design, patient populations, and VL measurement methods, has led to an absence of international consensus on LLV management. This study, following the WHO definition of LLV, included a large cohort of over 1,000 individuals to evaluate the prevalence of LLV and its association with VF. We found the global prevalence of LLV was 13.81%, with its occurrence associated with high baseline VL, AIDS-defining illness, and PI-based regimens at the initiation of ART. Moreover, LLV was strongly associated with an increased risk of VF and all-cause mortality.

Our findings confirmed a global prevalence of LLV, defined as 50–999 copies/mL, at 13.81%, with a range of 11.71–15.90%. This is consistent with previous studies. A study that reviewed articles based on different LLV definitions reported a prevalence range of 3– 26% [40]. We also performed a global analysis of the prevalence of LLV revealing that LLV prevalence in Africa was notably higher than that in other regions. An inadequate healthcare environment and limited drug availability, contributes to poor antiretroviral efficacy and drug adherence, that are key factors in the high prevalence of LLV. Other important challenges to HIV prevention and control including reduced funding, inequality issues, political and social barriers are also factors that cannot be ignored. Although we did observe a significant difference in LLV prevalence between high-income countries (HICs) and low- and middle-income countries (L/MICs), one study reported that LLV was more likely to occur in individuals from high-income settings [26], that was also associated with more frequent VL testing in HICs. In HICs, interventions such as adherence counselling, intensive surveillance, resistance testing, pharmacokinetic measurements, and ART regimen changes may have been implemented following the detection of an VL >50 copies/mL. Therefore, these interventions, however, may not be fully applicable to treatment programmes in L/MICs.

Our results demonstrated a significant correlation between LLV and VF. Additionally, pLLV was associated with VF, and this association, strengthened as VL increased. Although minor intermittent rebound in the VL is unlikely to result in pLLV or directly to VF, high-level blip levels still warrant attention. This suggests that the risk of VF escalates with the continuation of LLV, and higher VL levels correspond to a greater risk of VF. Aoko et al. also reported that, in a time-dependent regression model, LLV was related to VF, and this correlation increased with increasing VL [38], which is consistent with our results. High levels of LLV are also associated with other adverse clinical outcomes, including all-cause mortality, AIDS-related deaths, and serious non-AIDS events [12,30,41,42]. Remarkably, LLV results in an inability of PLWH to reconstitute a competent immune status associated with abnormal immune activation in these individuals [43]. Under pLLV conditions, cellular markers of immune activation remain consistently elevated. PLWH with pLLV exhibited higher CD8 ^+ ^T cell activation than that of virologically suppressed individuals [44]. The presence of LLV may generates new drug resistance mutations [45]. In another study, sequencing of 56 evaluable individuals revealed new resistance mutations in 37% of participants, with the most common being M184I/V, K103N, and M230L [46]. The high frequency of mutations inherent in reverse transcriptase and the lack of “error correction” during reverse transcription make HIV highly susceptible to mutations during high-speed replication [47,48]. The persistence of LLV provides a “breeding ground” for drug-resistant mutations. Thus, the presence of LLV increases the risk of HIV transmission and contributes to adverse clinical outcomes.

This study identified a VL ≥10^5^ copies/mL and AIDS-defining illness at baseline as risk factors for LLV. One potential cause of LLV is the reactivation of latently infected cells [43,44]. A higher baseline VL results in a larger viral reservoir and greater ability to discharge detectable viruses. Retrospective analysis demonstrated that newly diagnosed PLWH with VL ≥6 log10 copies/mL had a 2.2-fold increased risk of developing LLV before initiating ART [46]. Furthermore, AIDS-defined disease and CD4 < 200 cells/μL occur in PLWH with advanced AIDS, and the individuals may develop LLV due to complications or improper medication. Additionally, low baseline CD4^+^ levels have been reported to increase the risk of drug resistance and affect the occurrence of LLV [49]. In a prospective study based on the initial INSTI regimen, HIV RNA and CD4 were the decisive factors for virological non suppression [50]. This is consistent with our findings. Regardless of the CD4 count, ART should be initiated as soon as possible, which has long been included in major guidelines [13,14,16].

Based on the analysis of the initial ART regimen, we found that a PI-based regimen may increase the risk of LLV, consistent with previous studies [8,51,52]. In contrast, INSTI-and NNRTI-based regimens appeared to be protective factors. This is reflected in the Department of Health and Human Services guidelines, that discourage the use of unenhanced PIs as the initial regimens and instead recommend INSTI- and NNRTI-based regimens [2]. Although our results support this claim, different studies have reported differing opinions regarding whether NNRTIs reduce the incidence of LLV. Comparing PI to NNRTI, a prospective cohort study involving 1,511 PLWH across four African nations revealed an increased risk of LLV. The adjusted odds ratio value was as high as 4.06 (95% CI 2.20–7.48), especially in the 200–499 copies/mL group [8]. INSTI-based ART regimens are easier to administer via virological suppression. They had a shorter median time to achieve virological suppression than NNRTI- or PI-based ART regimens, according to a retrospective study of newly diagnosed HIV infections (the median time for the three groups was 137, 147, and 60 days, respectively; *p* < 0.01) [53]. Similarly, a recent study in an Australian cohort showed that PI-based regimens can increase the risk of VF. However, with the widespread use of INSTI, the incidence of VF has decreased from 9% in 2010 to 3% in 2021 [23]. Notably PIs are primarily used as second-line ART regimens for PLWH who fail first-line treatment, and because of their high drug resistance barrier, PIs are chosen by PLWH with poor immune function and drug resistance. Residual confounding factors may exist, as these individuals might be predisposed to failure regardless of the regimen used [8]. Our results suggest that NNRTIs protect against LLV; however, this remains a matter of debate. The initial ART regimens in the included cohorts were dominated by 2 NRTI +1 NNRTI and 2 NRTI +1PI [26,30,34,37]. Thus, our results indicate that 2 NRTI +1 NNRTI has a lower risk of LLV than 2 NRTI +1PI. In contrast, resistance mutations in LLV individuals were dominated by K103 N and V181 C/I/V [35], indicating that the resistance barrier for NNRTI is not high. Drug-resistant HIV strains are the result of dominant replication under the pressure of drug selectivity [54]. In PLWH receiving incomplete virological suppression therapy, the low resistance barrier of NNRTI is more likely to cause specific drug-resistance mutations in HIV. Additionally, there are restrictions on the use of high baseline VL in NNRTIs. Rilpivirine should not be used in PLWH with VL ≥10^5^ copies/mL. Chinese study shows that efavirenz is restricted to PLWH with VL ≥5 × 10^5^ copies/mL [55]. Coincidentally, our analysis results show that baseline VL ≥10^5^ copies/mL is the risk factor for LLV. However, whether NNRTIs protect against LLV remains controversial. Currently, no drugs can eliminate the HIV infection. INSTIs are recommended as the initial treatment for most PLWH because of their advantages such as safety, good tolerance, rapid viral suppression, potent antiviral activity, and a high genetic drug resistance barrier. Current guidelines recommend a single-tablet regimen comprising INSTIs as the preferred treatment option [14–16]. Our findings suggest that under the right circumstances, an INSTI-based regimen could serve as a switch to an ART regimen for PLWH with LLV.

We also found that NRTI resistance before treatment was a protective factor [37]. This is because a drug resistance test before ART initiation and optimization of the ART regimen, according to the test results, can significantly improve the therapeutic effect. According to a database analysis of PLWH in France, the drug resistance mutation rate in PLWH with LLV was far higher than that in individuals with continuous virological suppression [56]. Therefore, aggressive HIV drug resistance testing can help detect drug resistance mutations in a timely manner and enable precise ART, thus avoiding virological rebound due to primary drug resistance. Pre-treatment drug resistance tests guide doctors in creating accurate ART regimens, and the implementation of complete treatment monitoring plays an important role in helping PLWH achieve virological suppression as soon as possible.

This systematic review and meta-analysis included 16 large cohort studies with sample sizes of >1,000 individuals, 10 of which were multicentre studies. This allowed us to assess the strength of the associations between the exposure factors and outcomes more precisely, resulting in more reliable study results. All the cohort studies used uniform definitions of LLV and VF, which enabled us to combine and compare the results of different studies more accurately, and the findings from this meta-analysis are more likely to apply to a wider population. The studies were conducted in 15 countries on five continents, which provided a clearer picture of the prevalence of LLV in different countries and regions.

Our study had some limitations. First, the funnel plot analysis and Egger's test suggested that potential publication bias might have affected our pooled estimates. Lack of data on testing frequency and medication adherence may also have affected our results. In many studies, VL testing schedules, and the number and percentage of individuals missing medication have not been reported or adequately described. Additionally, the overall prevalence of LLV is highly heterogeneous across studies. This may be because of several other factors not considered in our analysis, such as whether ART was initiated promptly in the population, whether the initial ART regimen was determined based on the results of drug resistance testing, medication adherence in the population, psychosocial support, and comprehensive treatment. Given that data from most studies were unavailable, we could not include these variables in our analysis.

## Conclusion

This study demonstrated that despite active ART, PLWH continue to have remarkably high burden of LLV. There is a causal relationship between LLV and both an increased VF and all-cause mortality. Therefore, it is essential to emphasize the importance of screening for LLV risk factors prior to ART initiation. Frequent VL monitoring in PLWH is crucial for the timely detection of LLV and immediate intervention. Although no current drug can eradicate HIV, INSTIs are notable for their safer and efficacy, highlighting the need to redefine HIV treatment strategies and underscore the effectiveness of INSTI-based regimens.

## Supplementary Material

Supplementary Materials.docx

## Data Availability

All data generated or analyzed during this study are included in this published article.

## References

[1] UNAIDS. UNAIDS data 2020. (2020). [accessed 2023 Aug 13]. Available from: https://www.unaids.org/en/resources/documents/2020/unaids-data.

[2] DHHS. Panel on Antiretroviral Guidelines for Adults and Adolescents. Guidelines for the use of antiretroviral agents in adults and adolescents with HIV. (2024). [accessed 2024 Oct 13] Available from: https://clinicalinfo.hiv.gov/en/guidelines/adult-and-adolescent-arv.

[3] Rutstein SE, Hosseinipour MC, Kamwendo D, et al. Dried blood spots for viral load monitoring in Malawi: feasible and effective. PLoS One. 2015;10(4):e0124748. doi:10.1371/journal.pone.0124748 25898365 PMC4405546

[4] Drain PK, Dorward J, Bender A, et al. Point-of-Care HIV viral load testing: an essential tool for a sustainable global HIV/AIDS response. Clin Microbiol Rev. 2019;32(3):e00097–18. doi:10.1128/CMR.00097-18 31092508 PMC6589862

[5] Broyles LN, Luo R, Boeras D, et al. The risk of sexual transmission of HIV in individuals with low-level HIV viraemia: a systematic review. Lancet. 2023;402(10400):464–471. doi:10.1016/S0140-6736(23)00877-2 37490935 PMC10415671

[6] Vermeulen M, Lelie N, Coleman C, et al. Assessment of HIV transfusion transmission risk in South Africa: a 10-year analysis following implementation of individual donation nucleic acid amplification technology testing and donor demographics eligibility changes. Transfusion. 2019;59(1):267–276. doi:10.1111/trf.14959 30265757 PMC10419327

[7] WHO. New WHO guidance on HIV viral suppression and scientific updates released at IAS 2023. (2023). [accessed 2023 Aug 13]. Available from: https://www.who.int/news/item/23-07-2023-new-who-guidance-on-hiv-viral-suppression-and-scientific-updates-released-at-ias-2023.

[8] Esber A, Polyak C, Kiweewa F, et al. Persistent low-level viremia predicts subsequent virologic failure: Is it time to change the third 90? Clin Infect Dis. 2019;69(5):805–812. doi:10.1093/cid/ciy989 30462188

[9] Ioannidis JPA, Abrams EJ, Ammann A, et al. Perinatal transmission of human immunodeficiency virus type 1 by pregnant women with RNA virus loads<1000 copies/ml. J Infect Dis. 2001;183(4):539–545. doi:10.1086/318530 11170978

[10] Santoro MM, Fabeni L, Armenia D, et al. Reliability and clinical relevance of the HIV-1 drug resistance test in patients with low viremia levels. Clin Infect Dis. 2014;58(8):1156–1164. doi:10.1093/cid/ciu020 24429430

[11] Younas M, Psomas C, Reynes C, et al. Residual viremia is linked to a specific immune activation profile in HIV-1-infected adults under efficient antiretroviral therapy. Front Immunol. 2021;12:663843. doi:10.3389/fimmu.2021.663843 33859653 PMC8042152

[12] Quiros-Roldan E, Raffetti E, Castelli F, et al. Low-level viraemia, Measured as viraemia copy-years, as a prognostic factor for medium-long-term all-cause mortality: a MASTER cohort study. J Antimicrob Chemother. 2016;71(12):3519–3527. doi:10.1093/jac/dkw307 27543658

[13] WHO. HIV Prevention, Infant Diagnosis, Antiretroviral Initiation and Monitoring. (2021). [accessed 2023 Aug 13]. Available from: https://iris.who.int/bitstream/handle/10665/340190/9789240022232-eng.pdf?sequence = 1. 33822559

[14] DHHS. Guidelines for the Use of Antiretroviral Agents in Adults and Adolescents with HIV. (2023). [accessed 2023 Aug 13]. Available from: https://clinicalinfo.hiv.gov/en/guidelines/hiv-clinical-guidelines-adult-and-adolescent-arv/virologic-failure?view = full.

[15] Gandhi RT, Bedimo R, Hoy JF, et al. Antiretroviral drugs for treatment and prevention of HIV infection in adults: 2022 recommendations of the international antiviral society-USA panel. JAMA. 2023;329(1):63–84. doi:10.1001/jama.2022.22246 36454551

[16] EACS. Guidelines for treatment of people living with HIV version 12.0. (2023). [accessed 2023 Oct 18, 2023]. Available from: https://www.eacsociety.org/media/guidelines-12.0.pdf.

[17] Page MJ, McKenzie JE, Bossuyt PM, et al. The PRISMA 2020 statement: an updated guideline for reporting systematic reviews. Br Med J. 2021;29:n71. doi:10.1136/bmj.n71 PMC8005924 33782057

[18] Wells GA, Shea B, O'Connell D, et al. The Newcastle-Ottawa Scale (NOS) for assessing the quality of nonrandomised studies in meta-analyses. (2014). [accessed 2023 Aug 13, 2023]. Available from: https://www.ohri.ca/programs/clinical_epidemiology/oxford.asp.

[19] Sterne JAC, Harbord RM. Funnel plots in meta-analysis. Stata J. 2004;4(2):127–141. doi:10.1177/1536867X0400400204

[20] Harbord RM, Harris RJ, Sterne JAC. Updated tests for small-study effects in meta-analyses. Stata J. 2009;9(2):197–210. doi:10.1177/1536867X0900900202

[21] Elvstam O, Medstrand P, Yilmaz A, et al. Virological failure and all-cause mortality in HIV-positive adults with low-level viremia during antiretroviral treatment. PLoS One. 2017;12(7):e0180761. doi:10.1371/journal.pone.0180761 28683128 PMC5500364

[22] Zhang T, Ding H, An M, et al. Factors associated with high-risk low-level viremia leading to virologic failure: 16-year retrospective study of a Chinese antiretroviral therapy cohort. BMC Infect Dis. 2020;20(1):147. doi:10.1186/s12879-020-4837-y 32066392 PMC7026956

[23] Han WM, Broom J, Bopage R, et al. Investigating rates and predictors of viral blips, low-level viraemia and virological failure in the Australian HIV observational database. Trop Med Int Health. 2024;29(1):42–56. doi:10.1111/tmi.13951 38009461 PMC11108647

[24] Grennan JT, Loutfy MR, Su DS, et al. Magnitude of virologic blips is associated with a higher risk for virologic rebound in HIV-infected individuals: A recurrent events analysis. J Infect Dis. 2012;205(8):1230–1238. doi:10.1093/infdis/jis104 22438396 PMC3308904

[25] Laprise C, de Pokomandy A, Baril JG, et al. Virologic failure following persistent low-level viremia in a cohort of HIV-positive patients: results from 12 years of observation. Clin Infect Dis. 2013;57(10):1489–1496. doi:10.1093/cid/cit529 23946221

[26] Kanapathipillai R, McManus H, Cuong DD, et al. The significance of low-level viraemia in diverse settings: analysis of the treat Asia HIV observational database (TAHOD) and the Australian HIV observational database (AHOD). HIV Med. 2014;15(7):406–416. doi:10.1111/hiv.12124 24460817 PMC4107173

[27] Young J, Rickenbach M, Calmy A, et al. Transient detectable viremia and the risk of viral rebound in patients from the Swiss HIV Cohort Study. BMC Infect Dis. 2015;15:382. doi:10.1186/s12879-015-1120-8 26392270 PMC4578247

[28] Gonzalez-Serna A, Swenson LC, Watson B, et al. A single untimed plasma drug concentration measurement during low-level HIV viremia predicts virologic failure. Clin Microbiol Infect. 2016;22(12):1004.e9–1004.e16. doi:10.1016/j.cmi.2016.08.012 27585940

[29] Hermans LE, Moorhouse M, Carmona S, et al. Effect of HIV-1 low-level viraemia during antiretroviral therapy on treatment outcomes in WHO-guided South African treatment programmes: a multicentre cohort study. Lancet Infect Dis. 2018;18(2):188–197. doi:10.1016/S1473-3099(17)30681-3 29158101

[30] Elvstam O, Marrone G, Medstrand P, et al. All-Cause mortality and serious Non-AIDS events in adults With Low-level human immunodeficiency virus viremia during combination antiretroviral therapy: results from a Swedish nationwide observational study. Clin Infect Dis. 2021;72(12):2079–2086. doi:10.1093/cid/ciaa413 32271361 PMC8204776

[31] Li Q, Chen M, Zhao H, et al. Persistent Low-level viremia is an independent risk factor for virologic failure: A retrospective cohort study in China. Infect Drug Resist. 2021;14:4529–4537. doi:10.2147/IDR.S332924 34754201 PMC8572020

[32] Bai R, Lv S, Hua W, et al. Factors associated with human immunodeficiency virus-1 low-level viremia and its impact on virological and immunological outcomes: A retrospective cohort study in Beijing, China. HIV Med. 2022;23(Suppl 1):72–83. doi:10.1111/hiv.13251 35293102

[33] Chun HM, Abutu A, Milligan K, et al. Low-level viraemia among people living with HIV in Nigeria: a retrospective longitudinal cohort study. Lancet Glob Health. 2022;10(12):e1815–e1824. doi:10.1016/S2214-109X(22)00413-2 36400087 PMC9711923

[34] Ding H, Xu J, Liu J, et al. Outcomes of persistent low-level viremia among HIV patients on antiretroviral therapy: A prospective cohort study. HIV Med. 2022;23(Suppl 1):64–71. doi:10.1111/hiv.13250 35293103

[35] Liu P, You Y, Liao L, et al. Impact of low-level viremia with drug resistance on CD4 cell counts among people living with HIV on antiretroviral treatment in China. BMC Infect Dis. 2022;22(1):426. doi:10.1186/s12879-022-07417-z 35509014 PMC9066819

[36] Yu H, Yang Y, Cao D, et al. Association of low-level viremia with mortality among people living with HIV on antiretroviral therapy in dehong, Southwest China: A retrospective cohort study. HIV Med. 2023;24(1):37–45. doi:10.1111/hiv.13320 35578387

[37] Elvstam O, Malmborn K, Elén S, et al. Virologic failure following Low-level viremia and viral blips during antiretroviral therapy: results from a European multicenter cohort. Clin Infect Dis. 2023;76(1):25–31. doi:10.1093/cid/ciac762 36100984 PMC9825828

[38] Aoko A, Pals S, Ngugi T, et al. Retrospective longitudinal analysis of low-level viremia among HIV-1 infected adults on antiretroviral therapy in Kenya. E.Clin. Med. 2023;63:102166. doi:10.1016/j.eclinm.2023.102166 PMC10462863 37649807

[39] Liu J, Li C, Sun Y, et al. Characteristics of drug resistance mutations in ART-experienced HIV-1 patients with low-level viremia in Zhengzhou City, China. Sci Rep. 2024;14(1):10620. doi:10.1038/s41598-024-60965-z 38724547 PMC11082154

[40] WHO. Updated recommendations on HIV prevention, infant diagnosis, antiretroviral initiation and monitoring. (2021). [accessed 2023 Aug 13]. Available from: https://iris.who.int/bitstream/handle/10665/340190/9789240022232-eng.pdf?sequence = 1. 33822559

[41] Bernal E, Gómez JM, Jarrín I, et al. Low-Level viremia is associated with clinical progression in HIV-infected patients receiving antiretroviral treatment. J Acquir Immune Defic Syndr. 2018;78(3):329–337. doi:10.1097/QAI.0000000000001678 29543636

[42] Ganesan A, Hsieh HC, Chu X, et al. Low level viremia Is associated With serious non-AIDS events in people With HIV. Open Forum Infect Dis. 2024;11(4):ofae147. doi:10.1093/ofid/ofae147 38628953 PMC11020230

[43] Ostrowski SR, Katzenstein TL, Thim PT, et al. Low-level viremia and proviral DNA impede immune reconstitution in HIV-1-infected patients receiving highly active antiretroviral therapy. J Infect Dis. 2005;191(3):348–357. doi:10.1086/427340 15633093

[44] Karlsson AC, Younger SR, Martin JN, et al. Immunologic and virologic evolution during periods of intermittent and persistent low-level viremia. AIDS. 2004;18(7):981–989. doi:10.1097/00002030-200404300-00005 15096800

[45] Shu-Wei K, Zhuo-Hao L, Ting-Shu W, et al. Prevalence of drug resistance mutations in HIV-infected individuals with low-level viraemia under combination antiretroviral therapy: an observational study in a tertiary hospital in Northern Taiwan, 2017–19. J Antimicrob Chemother. 2021;76(3):722–728. doi:10.1093/jac/dkaa510 33331635

[46] Taiwo B, Gallien S, Aga E, et al. Antiretroviral drug resistance in HIV-1-infected patients experiencing persistent low-level viremia during first-line therapy. J Infect Dis. 2011;204(4):515–520. doi:10.1093/infdis/jir353 21791652 PMC3203388

[47] Domingo E, Sheldon J, Perales C. Viral quasispecies evolution. Microbiol Mol Biol Rev. 2012;76(2):159–216. doi:10.1128/MMBR.05023-11 22688811 PMC3372249

[48] Sallie R. Replicative homeostasis: a fundamental mechanism mediating selective viral replication and escape mutation. Virol J. 2005;2:10. doi:10.1186/1743-422X-2-10 15707489 PMC552327

[49] Charpentier C, Landman R, Laouénan C, et al. Persistent low-level HIV-1 RNA between 20 and 50 copies/mL in antiretroviral-treated patients: associated factors and virological outcome. J Antimicrob Chemother. 2012;67(9):2231–2235. doi:10.1093/jac/dks191 22643190

[50] Álvarez H, Mocroft A, Ryom L, et al. Plasma human immunodeficiency virus 1 RNA and CD4+ T-cell counts are determinants of virological nonsuppression outcomes With initial integrase inhibitor-based regimens: A prospective RESPOND cohort study. Clin Infect Dis. 2023;77(4):593–605. doi:10.1093/cid/ciad219 37052343 PMC10893964

[51] Ryscavage P, Kelly S, Li JZ, et al. Significance and clinical management of persistent low-level viremia and very-low-level viremia in HIV-1-infected patients. Antimicrob Agents Chemother. 2014;58(7):3585–3598. doi:10.1128/AAC.00076-14 24733471 PMC4068602

[52] Konstantopoulos C, Ribaudo H, Ragland K, et al. Antiretroviral regimen and suboptimal medication adherence are associated with low-level human immunodeficiency virus viremia. Open Forum Infect Dis. 2015;2(1):ofu119. doi:10.1093/ofid/ofu119 25884007 PMC4396432

[53] Jacobson K, Ogbuagu O. Integrase inhibitor-based regimens result in more rapid virologic suppression rates among treatment-naïve human immunodeficiency virus-infected patients compared to non-nucleoside and protease inhibitor-based regimens in a real-world clinical setting: A retrospective cohort study. Med. (Baltimore). 2018;97(43):e13016. doi:10.1097/MD.0000000000013016 PMC6221636 30412140

[54] Ndashimye E, Reyes PS, Arts EJ. New antiretroviral inhibitors and HIV-1 drug resistance: more focus on 90% HIV-1 isolates? FEMS Microbiol Rev. 2023;47(1):fuac040. doi:10.1093/femsre/fuac040 36130204 PMC9841967

[55] Chen S, Han Y, Song X-J, et al. Very high baseline HIV viremia impairs efficacy of non-nucleoside reverse transcriptase inhibitor-based ART: a long-term observation in treatment-naïve patients. Infect Dis Poverty. 2020;9(1):75. doi:10.1186/s40249-020-00700-8 32571409 PMC7310120

[56] Wirden M, Todesco E, Valantin M-A, et al. Low-level HIV-1 viraemia in patients on HAART: risk factors and management in clinical practice. J Antimicrob Chemother. 2015;70(8):2347–2353. doi:10.1093/jac/dkv099. 25921516

