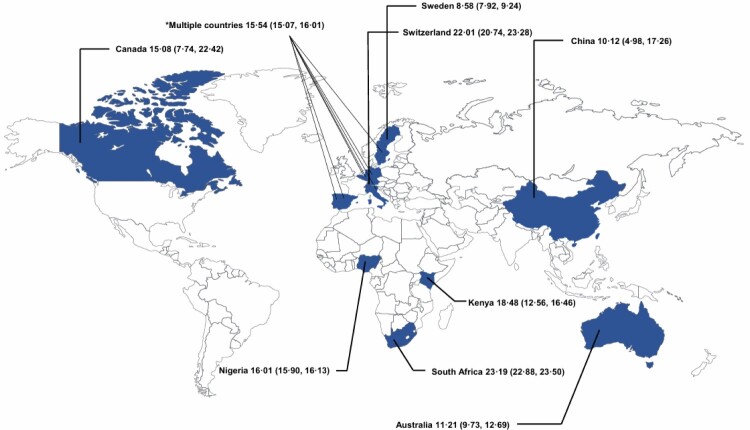# Correction

**DOI:** 10.1080/22221751.2025.2467547

**Published:** 2025-02-21

**Article title:** The Prevalence of Low-level Viraemia and Its Association with Virological Failure in People Living With HIV: A Systematic Review and Meta-Analysis

**Authors:** Zhao, S., Wang, W., Li, S., He, J., Duan, W., Fang, Z., Ma, X., Li, Z., Guo, C., Wang, W., Wu, H., Zhang, T., and HuangX.

**Journal:** *Emerging Microbes & Infections*

**Volume** 14 **Issue** 1

**DOI**: https://doi.org/10.1080/22221751.2024.2447613

When this article was first published online, the author mistakenly used incorrect map templates in Figure 3. The figure has now been corrected in the online version.

Wrong figure 3:

**Figure:**
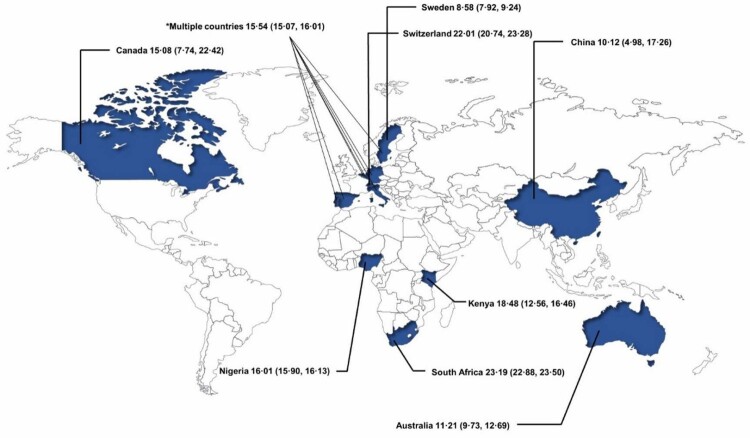

Correct Figure 3:

**Figure:**