# Supplementary material for: The prevalence of low-level viraemia and its association with virological failure in people living with HIV: a systematic review and meta-analysis
Source: Emerg Microbes Infect. 2024 Dec 27;14(1):2447613. doi: 10.1080/22221751.2024.2447613 (PMC11722027; doi:10.1080/22221751.2024.2447613)
Supplement: Supplementary Materials.docx [file TEMI_A_2447613_SM4691.docx]

**The Prevalence of Low-level Viraemia and Its Association with Virological Failure in People Living With HIV: A Systematic Review and Meta-Analysis**

**Supplementary appendix**

[Table S1 2](#_Toc172560485)

[Table S2. 3](#_Toc172560486)

[Table S3 8](#_Toc172560487)

[Table S4 10](#_Toc172560488)

[Figure S1 17](#_Toc172560489)

[Figure S2 18](#_Toc172560490)

[Figure S3 19](#_Toc172560491)

[Figure S4 20](#_Toc172560492)

[Figure S5 21](#_Toc172560493)

[Figure S6 22](#_Toc172560494)

[Figure S7 23](#_Toc172560495)

Table S1**. Search strategies**

**Search strategies for PubMed.**

| No. | Query | Results |
| --- | --- | --- |
| #1 | ((((((("HIV"[MeSH Terms]) OR ("HIV Infections"[MeSH Terms])) OR ("hiv infection"[Title/Abstract])) OR ("human immunodeficiency virus"[Title/Abstract])) OR ("aids virus"[Title/Abstract])) OR ("acquired immune deficiency syndrome virus"[Title/Abstract])) OR ("acquired immunodeficiency syndrome"[Title/Abstract])) OR ("HIV"[Title/Abstract]) | 461744 |
| #2 | (((((((("low-level viremia") OR ("low-level viraemia")) OR ("LLV")) OR ("low viral load")) OR ("LVL")) OR ("residual viraemia")) OR ("residual viremia")) OR ("viral blip")) OR (blip) | 2933 |
| #3 | #1 and #2 | 961 |

**Search strategies for Cochrane Library.**

| No. | Query | Results |
| --- | --- | --- |
| #1 | hiv:ti,ab,kw OR 'human immunodeficiency virus':ti,ab,kw OR 'aids virus':ti,ab,kw OR 'acquired immune deficiency syndrome virus':ti,ab,kw OR 'acquired immunodeficiency syndrome':ti,ab,kw | 33532 |
| #2 | MeSH descriptor: [HIV] explode all trees | 4224 |
| #3 | MeSH descriptor: [HIV infections] explode all trees | 17791 |
| #4 | #1 or #2 or #3 | 33663 |
| #5 | "low-level viremia" OR "low-level viraemia" OR LLV OR "low viral load" OR "LVL" OR "residual viraemia" OR "residual viremia" OR "virological failure" OR "viral blip" OR “blip” | 984 |
| #6 | #4 and #5 | 684 |

**Search strategies for Embase.**

| No. | Query | Results |
| --- | --- | --- |
| #1 | 'hiv infection':ti,ab,kw OR 'human immunodeficiency virus':ti,ab,kw OR 'aids virus':ti,ab,kw OR 'acquired immune deficiency syndrome virus':ti,ab,kw OR 'acquired immunodeficiency syndrome':ti,ab,kw OR hiv:ti,ab,kw | 507065 |
| #2 | 'human immunodeficiency virus'/exp | 224618 |
| #3 | 'human immunodeficiency virus infection'/exp | 827137 |
| #4 | #1 or #2 or #3 | 1015644 |
| #5 | 'low-level viremia' OR 'low-level viraemia' OR 'llv' OR 'low viral load' OR 'lvl' OR 'residual viraemia' OR 'residual viremia' OR 'viral blip' OR 'blip' | 4333 |
| #6 | #4 and #5 | 1555 |

**Search strategies for Web of Science.**

| No. | Query | Results |
| --- | --- | --- |
| #1 | (((((TS=("HIV")) OR TS=("Human Immunodeficiency Virus")) OR TS=("AIDS Virus")) OR TS=("Acquired Immune Deficiency Syndrome Virus")) OR TS=("Acquired Immunodeficiency Syndrome")) OR TS=("hiv infection") | 667499 |
| #2 | (((((((((TS=("low-level viremia")) OR TS=("low-level viraemia")) OR TS=("LLV")) OR TS=("low viral load")) OR TS=("LVL")) OR TS=("residual viraemia")) OR TS=("residual viremia")) OR TS=("virological failure")) OR TS=("viral blip")) OR TS=(blip) | 8874 |
| #3 | #1 and #2 | 5101 |
| #4 | (#1 and #2) AND (DT==(“Other” or “Meeting” or “Abstract” or “Letter” or “Case Report” or “Dissertation Thesis” or “Editorial Material” or “Review Article” or “Unspecified” or “Reference Material” or “News” or “Book” or “Biography” or “Data Paper” or “Retracted Publication” or “Retraction”)) | 1129 |

Table S2. **Cumulative summary characteristics of the studies included in the analysis.**

| **Study** | **Setting** | **Income level** | **Study design** | **Study period** | **Event/Total** | **LLV** | **Male** | **Age, years** | **Baseline CD4 count** | | **Baseline HIV VL** | **Initial ART regimen** | **Person-years of follow-up/Person-year incidence** |
| --- | --- | --- | --- | --- | --- | --- | --- | --- | --- | --- | --- | --- | --- |
|  |  |  |  |  |  |  |  |  | **Median (IQR)** | **<200 cells per μL** |  |  |  |
| Grennan et al 2012 | Canada | HICs | Retrospective cohort study | 2000.01.01-2009.01.05 | 756/3550 | blip | 2904 (82%) | 40 (34–47) | 190 (100–280) | 1815 patients (51%) | 4.9 log10 (4.3–5.1) | First-Line Therapy/Second-Line Therapy | NR |
| Laprise et al 2013 | Canada | HICs | Prospective cohort study | The date of HIV diagnosis varied from 1980 to 2012. | 165/1860 | pLLV | 1744 (93.8%) | 40.8 (35.4–46.9) | NR | NR | NR | First-Line Therapy/Second-Line Therapy | NR |
| Kanapathipillai et al 2014 | Australian | HICs | Prospective cohort study | AHOD-treatment after 1 January 1997 | 196/1748 | LLV | 1618 (92.6%) | NA | NR | 249 patients (14.2%) | NR | First-Line Therapy/Second-Line Therapy | 2.86/100 PYS (2.48–3.28) |
| Young et al 2015 | Switzerland | HICs | Retrospective cohort study | 1988–2014.05 | 901/4094 | blip | 73% | 40 | 430 | NR | NA | First-Line Therapy/Second-Line Therapy | NR |
| Gonzalez-Serna et al 2016 | Canada | HICs | Retrospective cohort study | 1999.09–2014.11 | 328/2176 | LLV | NR | NR | NR | NR | NR | First-Line Therapy/Second-Line Therapy | NR |
| Hermans et al 2017 | South Africa | L/MICs | Prospective cohort study | registered between Jan 1, 2007, and May 1, 2016, at 57 clinical sites | 16868/72740 | LLV | 23379 | First-line:35.7 (30.2–42.8);  Second-line: 35.7 (32.2–44.2) | First-line:186 (101–285);  Second-line: 228 (109–385) | First-line:32118 (55%);  Second-line: 1246 (44%) | NR | First-Line Therapy/Second-Line Therapy | First-line: 11.5/100 PYS (11.4–11.7);  Second-line:15.1/100 PYS (14.2–16.1) |
| Elvstam et al 2021 | Sweden | HICs | Retrospective cohort study | 1996.01–2017.06.14 | 597/6956 | blip | 4396 (63%) | 37 (31–45) | 240 (140–360) [18% missing] | NR | 73000 (18050–242000) [28% missing] | First-Line Therapy/Second-Line Therapy | 49986 PYS |
| Li et al 2021 | China | L/MICs | Retrospective cohort study | 2005.01.01–2018.12.31 | 427/8098 | LLV | 7635 (94.3%) | NA | 288 (160–389) | 2688 (33.2%) | 170051 (15562–135796) | First-Line Therapy/Second-Line Therapy | 0.96/100 PYS (0.94–0.99) |
| Bai et al 2022 | China | L/MICs | Retrospective cohort study | registered at hospital between January 1, 2005 and January 1, 2020 | 1113/10124 | LLV | First-line:8499 (96.7%);  Second-line: 1692 (90.5%) | First-line:31(27–40);  Second-line: 32 (27–40) | First-line:299.0 (189.8–416.0);  Second-line: 241.0 (92.0–373.3) | First-line:2339 (26.6%);  Second-line: 785 (42%) | First-line: 4.2log_10_ (3.6–4.8);  Second-line:4.4 log_10_ (3.8–5.0) | First-Line Therapy/Second-Line Therapy | NR |
| Chun et al 2022 | Nigeria | L/MICs | Retrospective cohort study | Initiated and received ART between Jan 1, 2016, and Sept 30, 2021 | 64480/402668 | LLV | 130524 (32.2%) | 34 (28–41) | NA | NA | NA | First-Line Therapy/Second-Line Therapy | NR |
| Ding et al 2022 | China | L/MICs | Retrospective cohort study | Between January 2011 and December 2018 | 81/1448* | pLLV | 1211 (94.0%) | 33 (27–44) | 252 (161–327) | 431 patients (33.5%) | 9 (7–13) | First-Line Therapy/Second-Line Therapy | 3915 PYS |
| Liu et al 2022 | China | L/MICs | Retrospective cohort study | Data from China’s national HIVDR surveillance database from Jan 1, 2008, to Dec 31, 2015 (data downloaded on Dec 31, 2017) | 1818/6530 | LLV | 4097 (62.7%) | 41 (34–49) | 340 (234–443) | 837 patients (12.8%) | 1.3 log_10_ (1.3–2.5) | First-Line Therapy/Second-Line Therapy | NR |
| Yu et al 2023 | China | L/MICs | Retrospective cohort study | Initiating ART in 2007–2016 and followed up to 31 December 2018 | 564/7273 | LLV | 4402 (60.5%) | 36 (30–43) | 254 (146–371) | 2638 patients (36.3%) | NR | First-Line Therapy/Second-Line Therapy | 45786.8 PYS |
| Aoko et al 2023 | Kenya | L/MICs | Retrospective cohort study | Data from the  National AIDs and STIs Control Programme (NASCOP)  VL database of PLHIV on ART between  January 2015 and December 2021. | 146690/ 793902 | LLV | 242,747 (30.6%) | 34.1 (27.5–42.5) | NR | NR | NR | First-Line Therapy/Second-Line Therapy | NR |
| Liu et al 2024 | China | L/MICs | Retrospective cohort study | PLWH who had experienced ART for  at least 6 months when visiting the Sixth People’s Hospital of Zhengzhou from January 2022 to August 2023 | 120/3616 | LLV | NR | NR | NR | NR | NR | First-Line Therapy/Second-Line Therapy | NR |

Abbreviations: LLV, low-level viraemia; VL, viral load; ART, antiretroviral therapy; IQR, interquartile range; HICs, high-income countries; L/MICs, low- and middle-income countries; NR, not reported; NA, non-available; PYS, person-years.

* We added the total number of subjects in this article to an additional 160 VF patients and deaths previously excluded. The baseline information was the data of 1288 no LLV and LLV patients included in the article.

Table S3**. Quality assessment of included studies.**

|  | **MINORS quality assessment tool of non-randomized studies** | | | | | | | | | | |
| --- | --- | --- | --- | --- | --- | --- | --- | --- | --- | --- | --- |
| **STUDY** | | **SELECTION** | | | | **COMPARABILITY** | **OUTCOME** | |  |  |  |
|  | | **Representativeness of the exposed cohort** | **Selection of the non-exposed cohort** | **Ascertainment of exposure** | **Demonstration that outcome of interest was not present at the start of study** | **Comparability of Cohorts on the Basis of the Design or Analysis Maximum: ☆☆** | **Assessment of outcome** | **Was follow-up long enough for outcomes to occur** | **Adequacy of follow-up of cohorts** | **SCORE** | **Evidence quality** |
| Grennan et al 2012 | | ☆ | ☆ | ☆ | ☆ | ☆☆ | ☆ | ☆ | ☆ | 9 | Low risk of bias |
| Laprise et al 2013 | | ☆ | ☆ | ☆ |  | ☆ | ☆ | ☆ | ☆ | 7 | High risk of bias |
| Kanapathipillai et al 2014 | | ☆ | ☆ | ☆ |  | ☆☆ | ☆ | ☆ | ☆ | 8 | Moderate risk of bias |
| Young et al 2015 | | ☆ | ☆ | ☆ |  | ☆ | ☆ | ☆ | ☆ | 7 | High risk of bias |
| Gonzalez-Serna et al 2016 | | ☆ | ☆ | ☆ | ☆ | ☆ | ☆ | ☆ | ☆ | 8 | Moderate risk of bias |
| Hermans et al 2017 | | ☆ | ☆ | ☆ | ☆ | ☆ | ☆ | ☆ | ☆ | 8 | Moderate risk of bias |
| Elvstam et al 2021 | | ☆ | ☆ | ☆ | ☆ | ☆☆ | ☆ | ☆ | ☆ | 9 | Low risk of bias |
| Li et al 2021 | | ☆ | ☆ | ☆ | ☆ | ☆ | ☆ | ☆ | ☆ | 8 | Moderate risk of bias |
| Bai et al 2022 | | ☆ | ☆ | ☆ | ☆ | ☆ | ☆ | ☆ | ☆ | 8 | Moderate risk of bias |
| Chun et al 2022 | | ☆ | ☆ | ☆ | ☆ | ☆ | ☆ | ☆ | ☆ | 8 | Moderate risk of bias |
| Ding et al 2022 | | ☆ | ☆ | ☆ | ☆ | ☆☆ | ☆ | ☆ | ☆ | 9 | Low risk of bias |
| Liu et al 2022 | | ☆ | ☆ | ☆ | ☆ | ☆ | ☆ | ☆ | ☆ | 8 | Moderate risk of bias |
| Yu et al 2023 | | ☆ | ☆ | ☆ | ☆ | ☆☆ | ☆ | ☆ | ☆ | 9 | Low risk of bias |
| Elvstam et al 2023 | | ☆ | ☆ | ☆ | ☆ | ☆☆ | ☆ | ☆ | ☆ | 9 | Low risk of bias |
| Aoko et al 2023 | | ☆ | ☆ | ☆ |  | ☆☆ | ☆ | ☆ | ☆ | 8 | Moderate risk of bias |
| Liu et al 2024 | | ☆ | ☆ | ☆ | ☆ | ☆ | ☆ | ☆ |  | 7 | High risk of bias |

Table S4**. Unadjusted and adjusted hazard risks associated with LLV**

| **Study** | **Participants** | **Unadjusted Analysis** | | **Adjusted Analysis** | | **Adjusted variables** |
| --- | --- | --- | --- | --- | --- | --- |
|  |  | **HR (95% CI)** | **P Value** | **HR (95% CI)** | **P Value** |  |
| **LLV vs. VF** | | | | | | |
| Grennan et al 2012 | blip 50–199 copies/mL | 0.93 (0.76–1.14) | 0.002 | 2.70 (1.44–5.06) | 0.002 | Assay type, age, sex, an HIV risk factor of injection drug use, year of cART initiation, type of cART regimen, and rate of HIV-1 RNA testing |
|  | blip 200–499 copies/mL | 1.13 (0.70–1.84) | 0.61 | 1.00 (0.53–1.88) | >0.99 |  |
|  | blip 500–999 copies/mL | 2.54 (1.40–4.62) | 0.47 | 1.08 (0.85–1.36) | 0.53 |  |
| Laprise et al 2013 | pLLV 50–199 copies/mL | 2.61 (1.88–3.63) | <0.001 | 2.22 (1.60–3.09) | <0.001 | Age, sex, date of HIV infection diagnosis, race, sexual orientation, monthly income, type of employment, CD4 cell count at baseline, injection drug use, and use of antiretroviral therapy. |
|  | pLLV 200–499 copies/mL | 2.92 (1.99–4.28) | <0.001 | 2.15 (1.46–3.17) | <0.001 |  |
|  | pLLV 500–999 copies/mL | 5.57 (3.67–8.46) | <0.001 | 4.85 (3.16–7.45) | <0.001 |  |
| Hermans et al 2017 | LLV 50–999 copies/mL /first-line cohort | NR | NR | 2.6 (2.5–2.8) | <0.0001 | Sex, age and CD4-cell count at ART initiation, and calendar year of ART initiation. This model included LLV as a dichotomous variable. |
|  | LLV 50–999 copies/mL /second-line cohort | NR | NR | 3.1 (2.5–4.0) | <0.0001 |  |
|  | LLV 50–199 copies/mL /first-line cohort | NR | NR | 1.9 (1.8–2.1) | <0.0001 | Sex, age and CD4-cell count at ART initiation, and calendar year of ART initiation. This model included LLV as a categorical variable. |
|  | LLV 200–399 copies/mL /first-line cohort | NR | NR | 3.2 (2.9–3.5) | <0.0001 |  |
|  | LLV 400–999 copies/mL /first-line cohort | NR | NR | 4.7 (4.2–5.2) | <0.0001 |  |
|  | LLV 50–199 copies/mL /second-line cohort | NR | NR | 2.1 (1.5–2.8) | <0.0001 |  |
|  | LLV 200–399 copies/mL /second-line cohort | NR | NR | 3.7 (2.6–5.4) | <0.0001 |  |
|  | LLV 400–999 copies/mL /second-line cohort | NR | NR | 6.8 (4.7–9.8) | <0.0001 |  |
| Li et al 2021 | blip 50–200 copies/mL | 0.80 (0.42–1.50) | 0.487 | 0.73 (0.39–1.39) | 0.341 | Age, gender, HIV transmission route, marital status, HBV/HCV infection, ART regimen, HIV-1 RNA VL, and CD4^+^ cell count at baseline. |
|  | blip 201–400 copies/mL | 1.23 (0.31–4.95) | 0.769 | 1.41 (0.35–5.69) | 0.629 |  |
|  | blip 401–999 copies/mL | 0.96 (0.14–6.84) | 0.967 | 0.97 (0.13–6.91) | 0.972 |  |
|  | pLLV 50–200 copies/mL | 5.38 (2.77–10.46) | <0.001 | 4.12 (2.16–7.84) | <0.001 |  |
|  | pLLV 201–400 copies/mL | 7.54 (3.35–16.98) | <0.001 | 4.96 (2.17–11.32) | <0.001 |  |
|  | pLLV 401–999 copies/mL | 15.78 (9.78–25.46) | <0.001 | 15.03 (9.42–23.98) | <0.001 |  |
| Ding et al 2022 | pLLV 50–999 copies/mL | NR | NR | 8.01 (3.80–16.90) | NR | Sex, age, HIV risk factors, race, date of ART start, regimen at the baseline, HBV, HCV, CD4 count and HIV-1 RNA at ART start. |
|  | pLLV 50–199 copies/mL | NR | NR | 3.10 (0.86–11.09) | NR |  |
|  | pLLV 200–499 copies/mL | NR | NR | 14.92 (5.92–37.60) | NR |  |
|  | pLLV 500–999 copies/mL | NR | NR | 13.68 (3.61–51.87) | NR |  |
| Elvstam et al 2023 | blip 50–999 copies/mL | 1.4 (1.2–1.7) | NR | 1.7 (1.3–2.2) | NR | Age (modelled linearly), sex (male/female), CD4 count (modelled linearly, time-updated), VL before start of ART (modelled logarithmically), transmission group (injecting drug use [IDU]/non-IDU), subtype (A/B/C/other), regimen type (NNRTI-based/PI-based/INSTI-based/other), and treatment experience. |
|  | pLLV 50–199 copies/mL | 2.6 (2.3–3.1) | NR | 2.2 (1.6–3.0) | NR |  |
| **LLV vs. all-cause mortality** | | | | | | |
| Elvstam et al 2021 | pLLV 50–999 copies/mL | 1.7 (1.2–2.4) | NR | 2.2 (1.3–3.6) | NR | Sex, age (defined as age at outcome event), CD4 count and VL before initiation of any ART, birth in Sweden, injection drug use, exposure to antiretroviral drugs prior to cART, and documented treatment interruptions during follow-up. |
|  | pLLV 50–199 copies/mL | 2.3 (1.5–3.3) | NR | 2.2 (1.3–3.8) | NR |  |
|  | pLLV 200–999 copies/mL | 1.2 (0.68–2.0) | NR | 2.1 (0.96–4.7) | NR |  |
| Yu et al 2023 | LLV 50–199 copies/mL | 1.06 (0.73–1.54) | NR | 1.00 (0.68–1.45) | NR | Time-varying VL groups, age, gender, education level, transmission route, OIs, CD4 cell count, haemoglobin (99 participants with missing values of baseline haemoglobin were not included due to potential information bias), HBsAg and antiretroviral regimen at baseline. |
|  | LLV 200–999 copies/mL | 1.75 (1.17–2.60) | NR | 1.56 (1.04–2.32) | NR |  |
| **LLV vs. AIDS-related death** | | | | | | |
| Elvstam et al 2021 | pLLV 50–999 copies/mL | 0.45 (0.11–1.9) | NR | No event | NR | Sex, age (defined as age at outcome event), CD4 count and VL before initiation of any ART, birth in Sweden, injection drug use, exposure to antiretroviral drugs prior to cART, and documented treatment interruptions during follow-up. |
| Yu et al 2023 | LLV 50–199 copies/mL | 1.29 (0.71–2.34) | NR | 1.12 (0.62–2.04) | NR | Time-varying VL groups, age, gender, education level, transmission route, OIs, CD4 cell count, haemoglobin, HBsAg and antiretroviral regimen at baseline. |
|  | LLV 200–999 copies/mL | 2.85 (1.64–4.96) | NR | 2.37 (1.36–4.14) | NR |  |
| **LLV vs. NAEs** | | | | | | |
| Elvstam et al 2021 | pLLV 50–999 copies/mL | 1.2 (0.92–1.6) | NR | 1.2 (0.78–1.8) | NR | Sex, age (defined as age at outcome event), CD4 count and VL before initiation of any ART, birth in Sweden, injection drug use, exposure to antiretroviral drugs prior to cART, and documented treatment interruptions during follow-up. |
|  | pLLV 50–199 copies/mL | 1.1 (0.76–1.6) | NR | 0.86 (0.50–1.5) | NR |  |
|  | pLLV 200–999 copies/mL | 1.3 (0.92–1.9) | NR | 2.0 (1.2–3.6) | NR |  |
| Ding et al 2022 | pLLV 50–999 copies/mL | NR | NR | 8.39 (4.07–17.30) | NR | Sex, age, HIV risk factors, race, date of ART start, regimen at the baseline, HBV, HCV, CD4 count and HIV-1 RNA at ART start. |
|  | pLLV 50–199 copies/mL | NR | NR | 4.55 (1.80–11.53) | NR |  |
|  | pLLV 200–499 copies/mL | NR | NR | 19.14 (6.72–54.52) | NR |  |
|  | pLLV 500–999 copies/mL | NR | NR | 19.22 (4.92–75.05) | NR |  |

Abbreviations: HR, hazard risk; CI, confidence interval; LLV, low-level viraemia; VF, virological suppression; pLLV, persistence low-level viraemia; NR, not reported; ART, antiretroviral therapy; VL, viral load; HBV, hepatitis B virus; HCV, hepatitis C virus; IDU, injecting drug use; NNRTI, nonnucleoside reverse transcriptase inhibitor; PI, protease inhibitor; INSTI, integrase inhibitor; OI, opportunistic infection; HBsAg, hepatitis B surface antigen; AIDS, acquired immunodeficiency syndrome; NAEs, non-AIDS events.

A

**
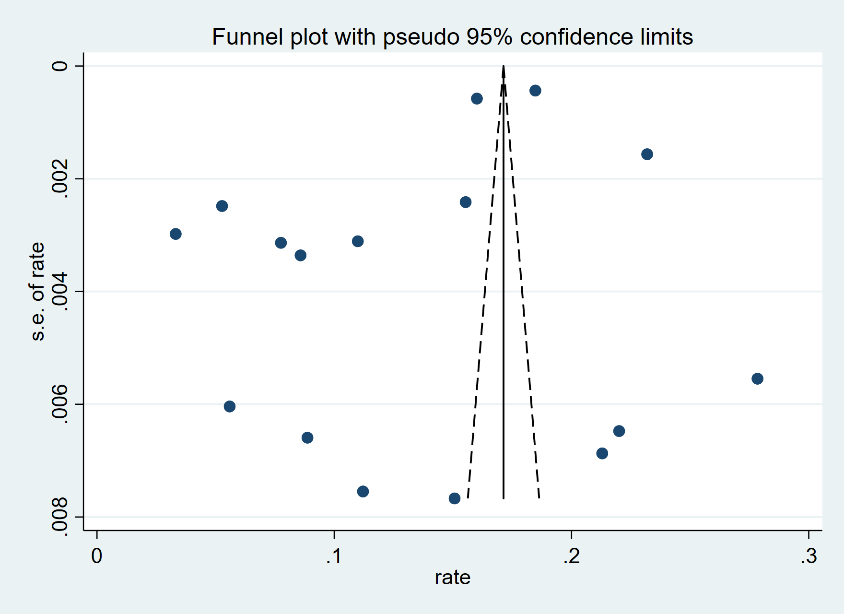
**

B

**
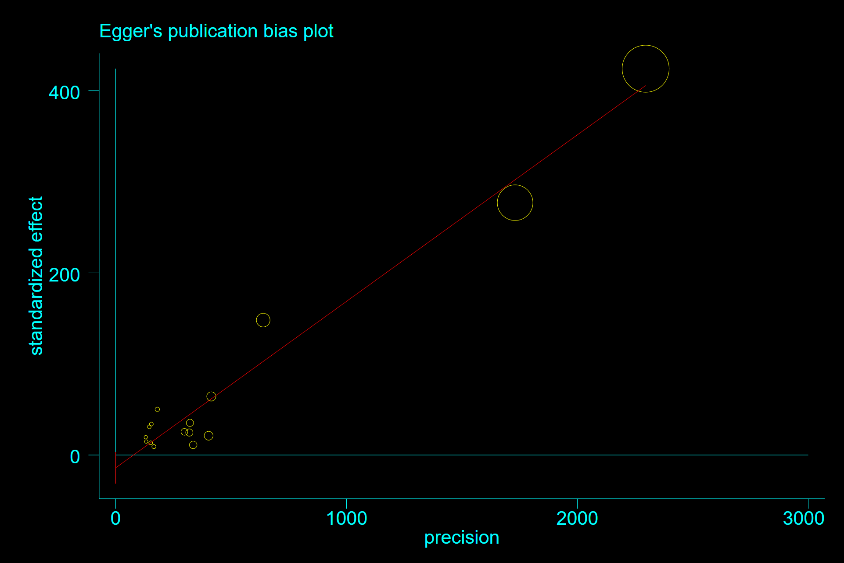
**

C

**
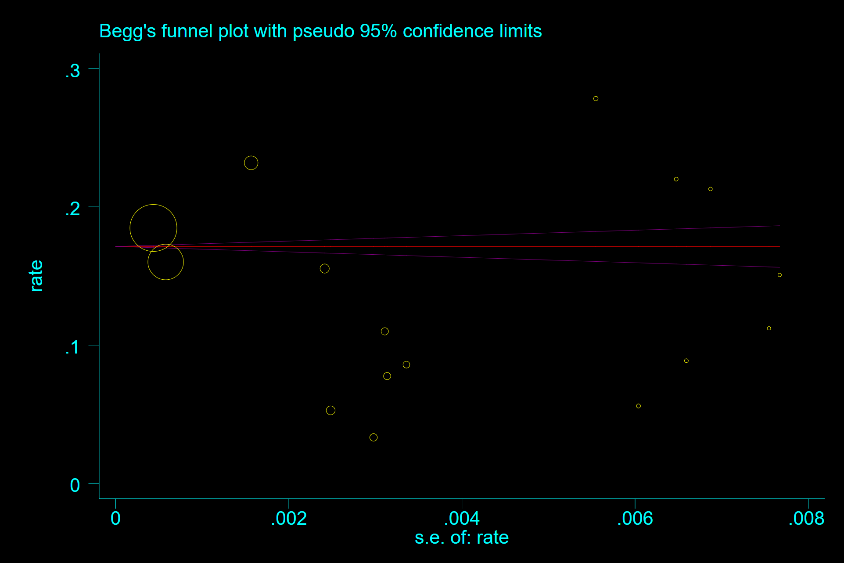
**

Figure S1**.** **Publication bias test by funnel plot (A), egger`s test (B), and begg`s test (C).**

**
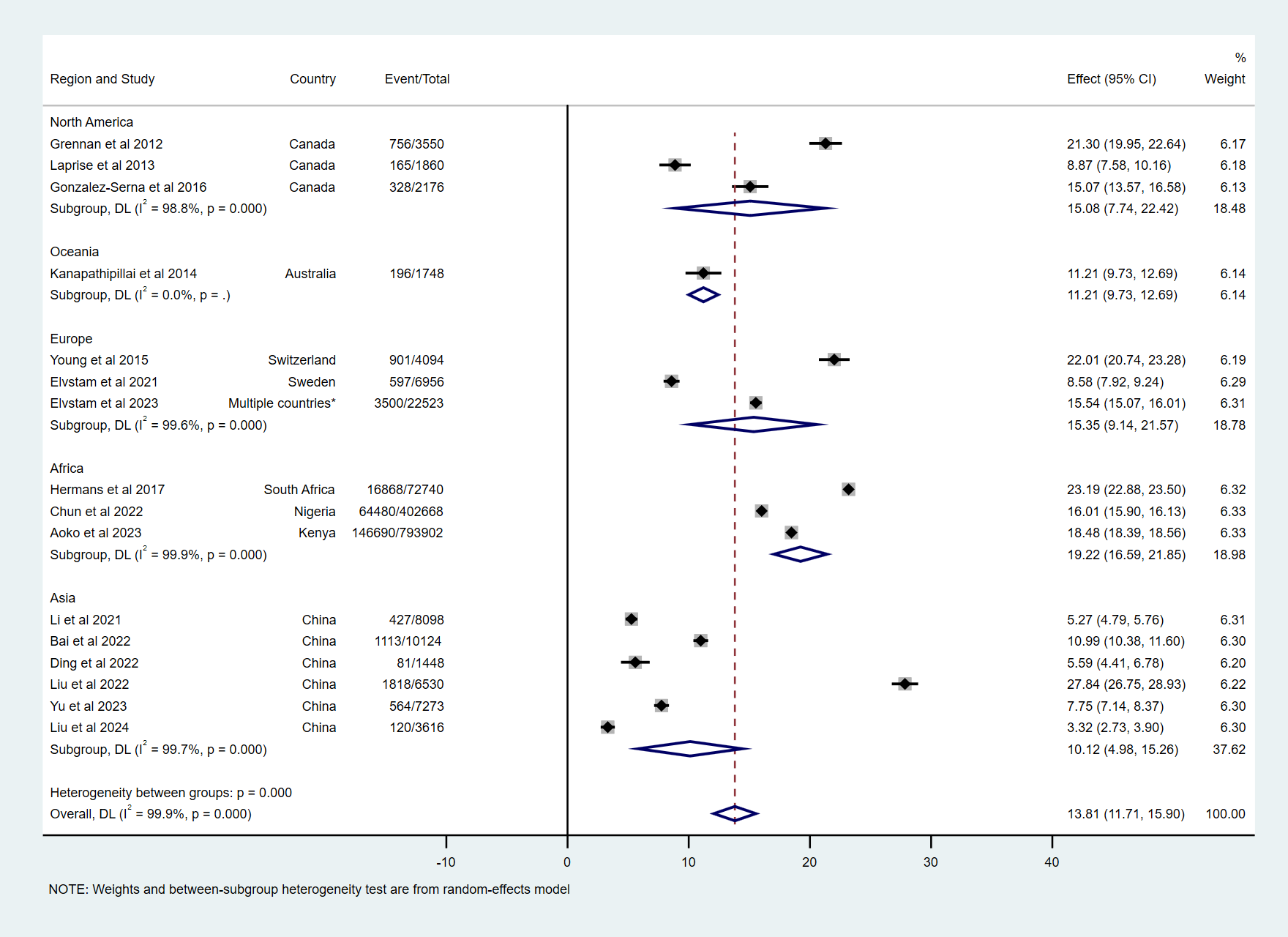
**

Figure S2**.** **Forest plot of LLV among PLWH subgrouped by region.** Abbreviations: CI, confidence interval, LLV, low-level viraemia.

*Multiple countries: Spain, Italy, Sweden, Germany, Luxembourg, Portugal, and Belgium.

A

**
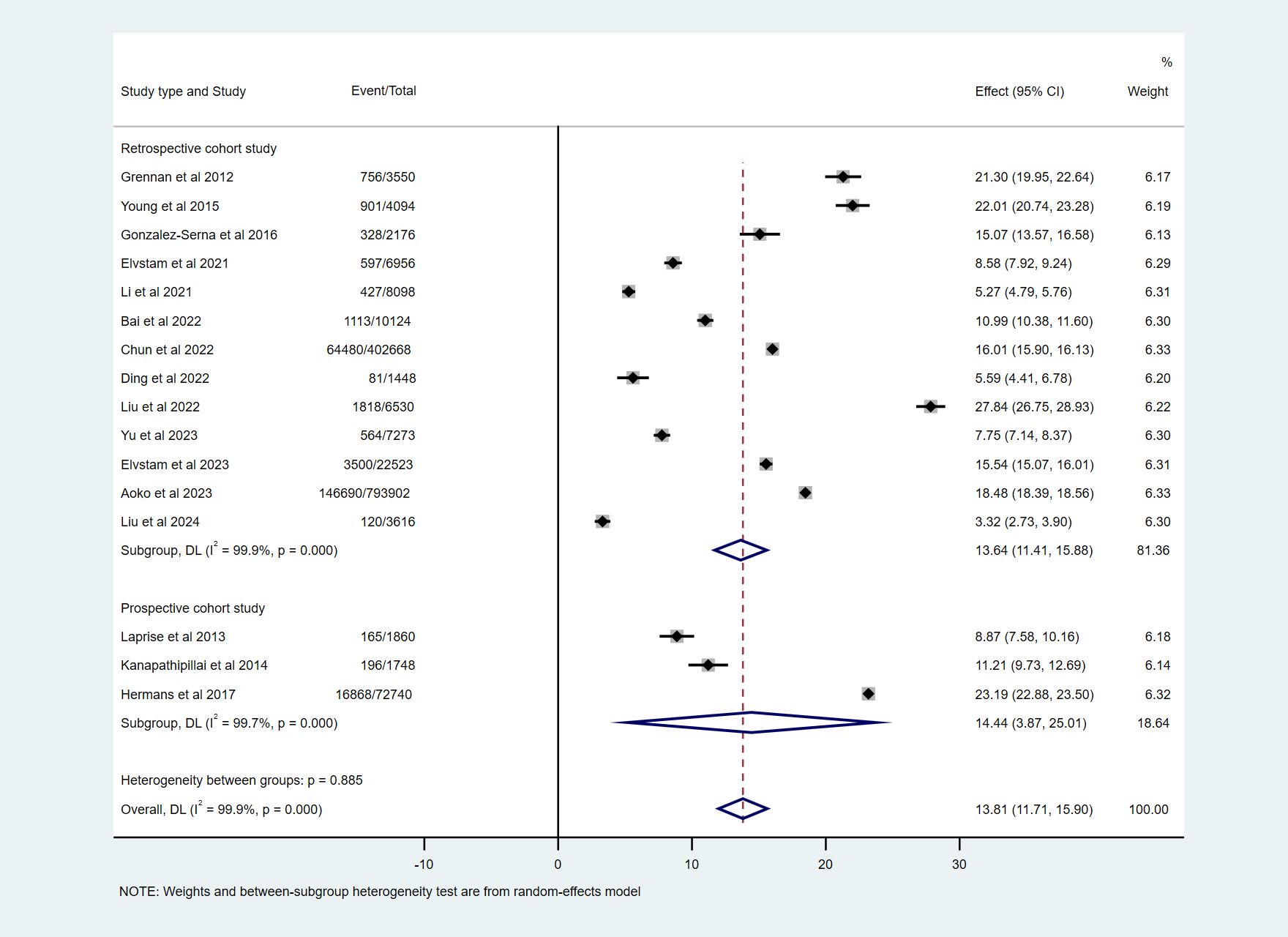
**

B

**
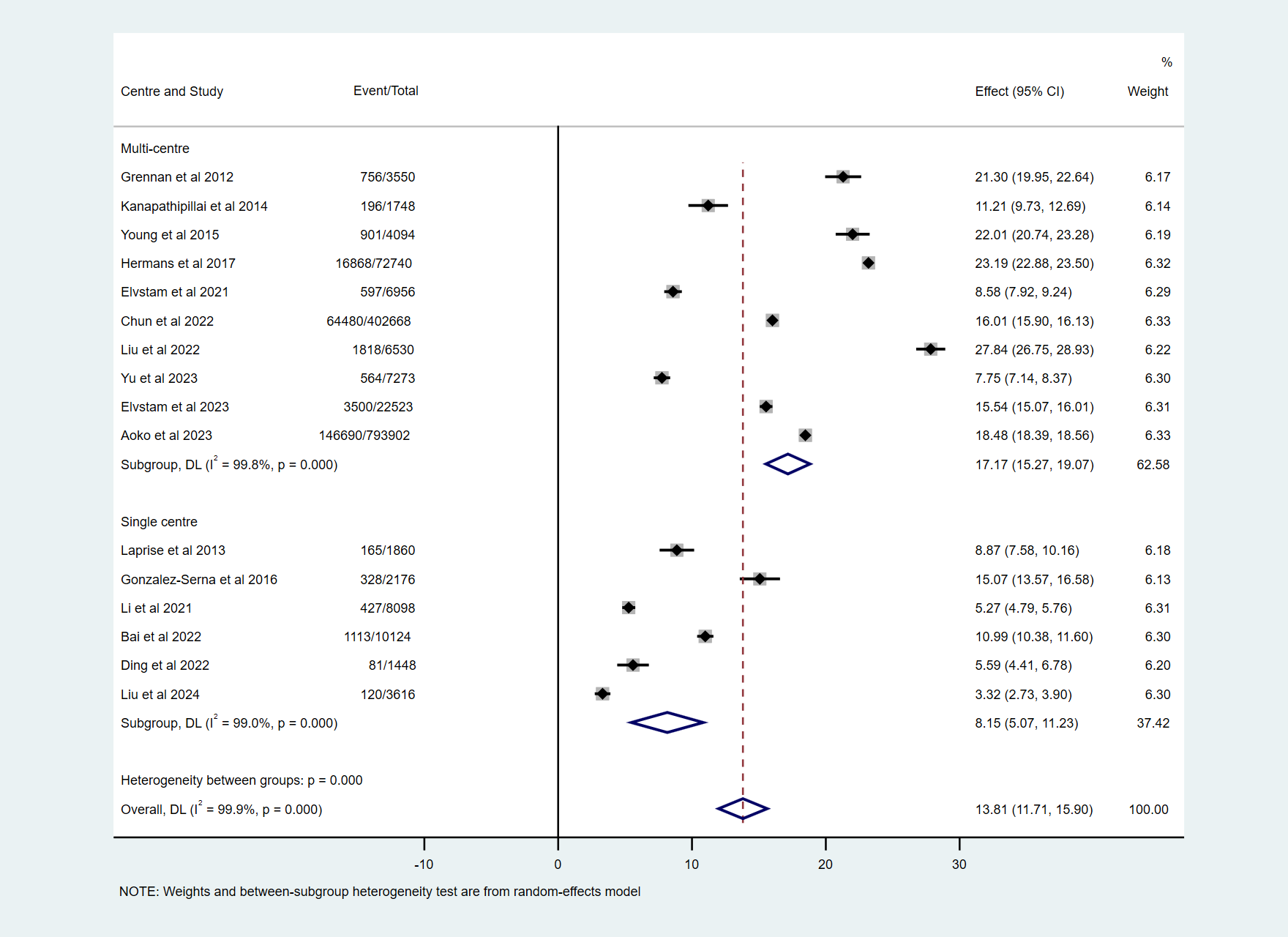
**

C

**
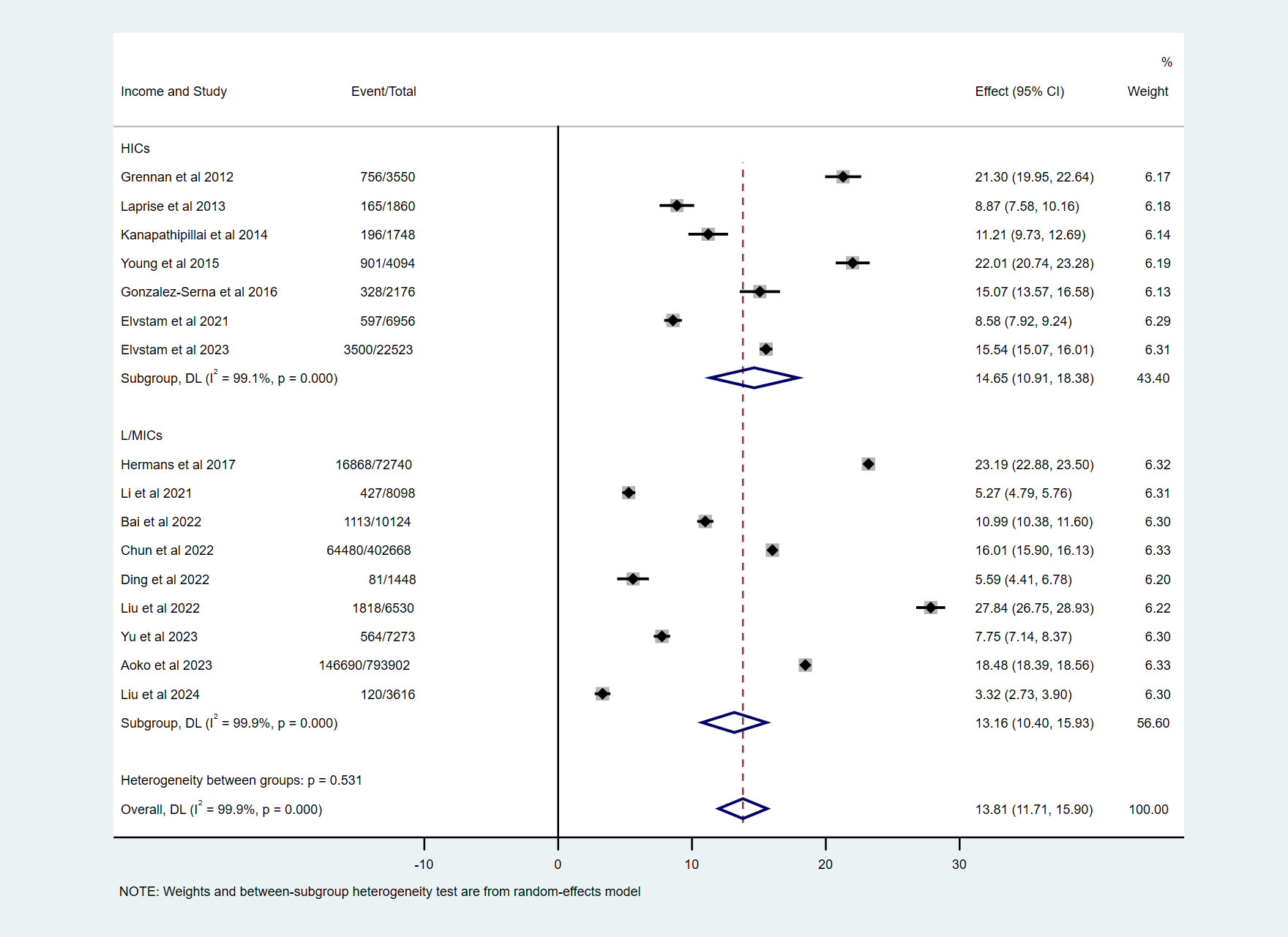
**

Figure S3**. LLV prevalence rate by study type (A), study site (B), and income (C).** Abbreviations: LLV, low-level viraemia; CI, confidence interval; HICs, high-income countries; L/MICs, low- and middle-income countries.

* Multicenter represents simultaneous clinical trials of the same trial program at different sites and units from the same country or different countries.

Figure S4**.** **Pooled aHR for assessing the association between the risk of VF and LLV.** Abbreviations: aHR, adjusted hazard ratio; CI, confidence interval; LLV, low-level viraemia; pLLV, persistence low-level viraemia; VL, viral load.

Li et al. `s standard for stratified LLV is different from other articles.

**A B C**

Figure S5**. Pooled unadjusted HR for assessing the association between the risk of VF and LLV (A) subgrouped by blip (B) and pLLV (C).** Abbreviations: HR, hazard ratio; CI, confidence interval; VL, viral load; pLLV, persistence low-level viraemia.

Li et al. `s standard for stratified LLV is different from other articles.

**
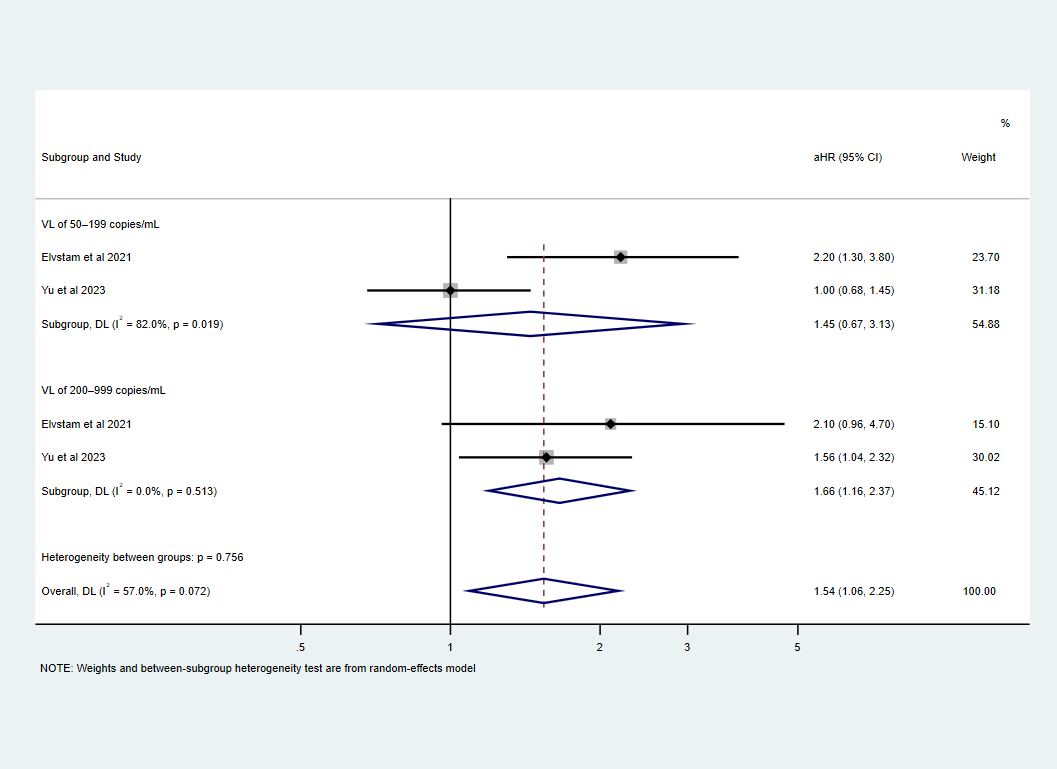
**

Figure S6. **Association between LLV and all-cause mortality.** Abbreviations: aHR, adjusted hazard ratio; CI, confidence interval; VL, viral load; LLV, low-level viraemia.

Figure S7**. Forest plot displaying risk factors of LLV by sex (A), route of HIV acquisition (B), baseline characteristic (C) and ART status (D).** Abbreviations: RR, risk ratio; CI, confidence interval; ART, antiretroviral therapy; IDU, injecting drug use; HBsAg, hepatitis B surface antigen; HCV, hepatitis C virus; NRTI, nucleoside reverse transcriptase inhibitor; LLV, low-level viraemia.
